# Triple-Negative Breast Cancer Progression and Drug Resistance in the Context of Epithelial–Mesenchymal Transition

**DOI:** 10.3390/cancers17020228

**Published:** 2025-01-12

**Authors:** Ewa Błaszczak, Paulina Miziak, Adrian Odrzywolski, Marzena Baran, Ewelina Gumbarewicz, Andrzej Stepulak

**Affiliations:** Department of Biochemistry and Molecular Biology, Medical University of Lublin, 1 Chodzki Street, 20-093 Lublin, Poland

**Keywords:** triple-negative breast cancer, TNBC, resistance, chemoresistance, epithelial–mesenchymal transition, EMT, EMT markers, treatment

## Abstract

Triple-negative breast cancer (TNBC) is one of the most difficult subtypes of breast cancer to treat due to its distinct clinical and molecular characteristics. Patients with TNBC face a high recurrence rate, an increased risk of metastasis, and lower overall survival compared to other breast cancer subtypes. Despite advancements in targeted therapies, traditional chemotherapy (primarily using platinum compounds and taxanes) continues to be the standard treatment for TNBC, often with limited long-term efficacy. TNBC tumors are heterogeneous, displaying a diverse mutation profile and considerable chromosomal instability, which complicates therapeutic interventions. The development of chemoresistance in TNBC is frequently associated with the process of epithelial–mesenchymal transition (EMT), during which epithelial tumor cells acquire a mesenchymal-like phenotype. This shift enhances metastatic potential, while simultaneously reducing the effectiveness of standard chemotherapeutics. It has also been suggested that EMT plays a central role in the development of cancer stem cells. Hence, there is growing interest in exploring small-molecule inhibitors that target the EMT process as a future strategy for overcoming resistance and improving outcomes for patients with TNBC. This review focuses on the progression and drug resistance of TNBC with an emphasis on the role of EMT in these processes. We present TNBC-specific and EMT-related molecular features, key EMT protein markers, and various signaling pathways involved. We also discuss other important mechanisms and factors related to chemoresistance in TNBC within the context of EMT, highlighting treatment advancements to improve patients’ outcomes.

## Simple Summary

Triple-negative breast cancer (TNBC) is a challenging-to-treat breast cancer subtype, characterized by high recurrence, increased risk of metastasis, and lower survival for patients compared to other breast cancer subtypes. Depending on the clinical advancement of the disease, surgery, radiotherapy, and chemotherapy remain the standard treatment, though they often have limited long-term efficacy. Chemoresistance in TNBC is closely related to the epithelial–mesenchymal transition (EMT), a process where tumor cells gain mesenchymal-like characteristics. This, in turn, increases their metastatic potential and resistance to standard chemotherapeutic treatments. There is a growing interest in small-molecule inhibitors targeting EMT as a potential strategy to overcome resistance and improve TNBC patient outcomes. This review discusses the TNBC progression and drug resistance within the context of EMT, highlighting molecular features, key EMT protein markers, and signaling pathways. It also explores other mechanisms and factors related to chemoresistance in TNBC with an emphasis on treatment advancements.

## 1. Introduction

According to Cancer Statistics, breast cancer (BC) is the most frequently diagnosed cancer, accounting for 32% of new cancer cases in 2024. Although significant progress has been made in the early detection and treatment of BC, it is still the primary cause of cancer-related mortality among females [1]. This clinically heterogeneous disease includes several distinct molecular subtypes, with the four most common being hormone receptor-positive (HR-positive) luminal A and luminal B, human epidermal growth factor receptor 2-positive (HER2-positive), and triple-negative breast cancer (TNBC). The luminal A subtype is defined as estrogen receptor (ER)/progesterone receptor (PR)-positive, HER2-negative, and Ki-67 protein expression index below 20%, reflecting the immunohistochemical staining outcomes for the patient samples. Ki-67 is a protein present in all active phases of the cell cycle but absent in the quiescent G0 phase. Hence, the level of Ki-67 expression reflects the proliferative activity of cancer cells and serves as the prognostic biomarker in BC [2]. High Ki-67 expression, defined as >30%, is associated with poorer prognosis in terms of disease-free survival (DFS) and overall survival (OS) of patients with TNBC [3]. High Ki-67 labeling index (LI) in the studied group of 52 TNBC patients was strongly associated with the presence of lymph node metastases, elevated tumor grade, advanced clinical stage, poor patient outcomes, and an inability to achieve pathological complete response (pCR) [4].

**Figure 1:**
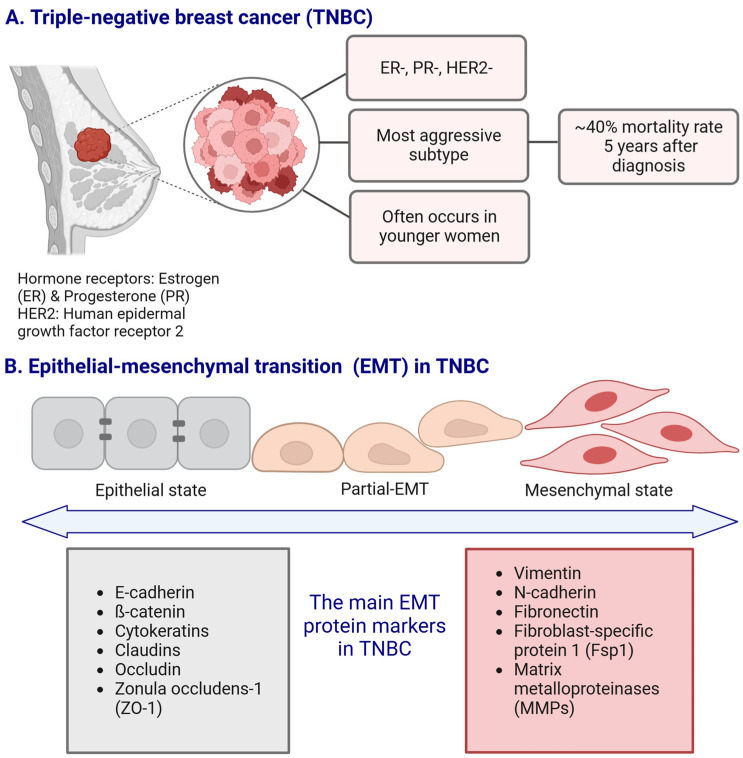
The main characteristics of the triple-negative breast cancer (TNBC) subtype ((**A**); panel (**A**) adapted from [25] with modifications), and the schematic representation of the epithelial–mesenchymal transition (EMT) with the main EMT protein markers associated with TNBC (**B**). Created in https://BioRender.com.

The luminal B subtype is ER/PR-positive (that may also lack the expression of PR), HER2-negative, and Ki-67 protein expression index ≥ 20%. HER2-positive subtype is characterized by HER2 overexpression but a lack of ER and PR receptors, while TNBC is marked by the absence of hormone receptors and HER2 expression (Figure 1A) [5,6]. Each molecular subtype of TNBC itself displays unique biological features [7,8,9] that contribute to its clinical behavior and therapeutic response. These subtypes are distinguished by specific molecular signatures, such as gene expression patterns, signaling pathway activation, and immune response regulation, which impact their aggressiveness, metastatic potential, and sensitivity to targeted treatments [9,10].

TNBC is a highly aggressive BC subtype, with its incidence steadily increasing. It is associated with the worst prognosis and a significantly shorter patient survival time compared to other BC subtypes [11,12]. Epidemiological and population-based studies indicate that TNBC primarily affects women under the age of 40 [13,14] and represents approximately 25% of all breast cancer cases [15]. The highest likelihood of TNBC recurrence occurs within the first three years, with a large number of deaths taking place within the first five years following the initial treatment [16]. The mortality rate for TNBC patients is approximately 40% within the first five years of diagnosis. The mortality rate within three months following TNBC recurrence can reach as high as 75%, reflecting the aggressive and treatment-resistant nature of the disease [17].

TNBC is characterized by a lack of effective treatment options. This is primarily due to the absence of ER, PR, and HER2 expression, which renders targeted and endocrine therapies ineffective. As a result, chemotherapy remains the primary therapeutic strategy for managing TNBC. In recent years, neoadjuvant chemotherapy (NACT) regimens, i.e., chemotherapy treatments administered before surgery, have become particularly important for treating TNBC [17]. These regimens aim to decrease the tumor size, making surgical removal of any remaining tumor tissue more feasible and less invasive [18]. It has been reported that TNBC patients achieve significantly higher rates of pCR with neoadjuvant chemotherapy compared to HR-positive BCs. This improved pCR is closely associated with better long-term outcomes, including reduced recurrence rates and enhanced survival [19]. Despite these, TNBC management faces challenges, especially related to treatment resistance. About half of the patients with localized TNBC who undergo NACT still have significant residual cancer burden (RCB-II or -III) as identified through the histopathological assessment of the breast tissue and axillary lymph nodes following surgery. This limited response to chemotherapy correlates with a 40–80% likelihood of disease recurrence, leading to distant metastasis and, for many patients, eventual death [20]. The introduction of immune checkpoint inhibitors (ICIs) made advancements in immunotherapy for TNBC. However, current evidence suggests that monotherapy with PD-1 (programmed cell death-1) or its ligand (PD-L1) inhibitors shows limited effectiveness, with only a small subset of patients experiencing substantial benefits [21]. These highlight an urgent need for deciphering molecular mechanisms related to TNBC progression and drug resistance to develop novel therapeutic approaches.

Epithelial–mesenchymal transition (EMT) is a process that promotes the detachment of cancer cells from the primary tumor, increasing their migratory and invasive potential, which makes them more likely to spread to surrounding tissues [22]. This transition contributes to TNBC’s aggressive nature and its resistance to conventional therapies [23], highlighting the importance of targeting EMT-related pathways for improving treatment strategies. The EMT process induced by signals from the tumor microenvironment (TME), such as growth factors, cytokines, and hypoxia, enables tumor cells to acquire mesenchymal characteristics. These changes include the loss of cell junction proteins, altered cell polarity, and enhanced stem cell properties, alongside cytoskeletal reorganization. As EMT progresses, overall mesenchymal markers (Figure 1B) such as vimentin and N-cadherin are upregulated, while the expression of epithelial markers such as E-cadherin decreases, leading to increased cancer cell motility and invasion (recently summarized by us in [24]).

Here, we discuss the current understanding of the TNBC progression and drug resistance within the context of the EMT process. Based on The Cancer Genome Atlas (TCGA) database (URL: https://www.cancer.gov/ccg/research/genome-sequencing/tcga (accessed on 16 November 2024)), we first analyze the frequency of the major somatic mutations in TNBC compared to other BC subtypes to briefly introduce the general, molecular landscape, as well as the expression levels of genes related to EMT in TNBC compared to other BC subtypes. We also present key EMT protein markers with an emphasis on TNBC progression. Moreover, we highlight the molecular mechanisms and signaling pathways associated with EMT in TNBC, particularly in relation to drug resistance and disease progression. Other mechanisms contributing to TNBC chemoresistance are also reviewed. A comprehensive understanding of molecular mechanisms and signaling pathways linked to the EMT process may help in the development of novel anti-cancer therapies for this highly aggressive and treatment-resistant BC subtype.

## 2. Brief Overview of Molecular Landscape of Triple-Negative Breast Cancer (TNBC)

Lehmann et al. [26] proposed six distinct subtypes of TNBC based on gene expression profiling from 21 BC datasets and 587 TNBC patients’ samples. These are two basal-like subtypes (BL1 and BL2), luminal androgen receptor (LAR), immunomodulatory (IM), mesenchymal (M), and mesenchymal stem-like (MSL). BL1 is characterized by DNA repair and cell-cycle gene signatures, while BL2 shows enrichment in growth factor signaling and myoepithelial differentiation genes. The LAR subtype is linked to androgen receptor (AR) signaling. The IM subtype is linked to immune response regulation, and both M and MSL are enriched in genes associated with cell motility and EMT [27,28,29]. Burstein et al. [30]. categorized TNBC from 198 patients into four subtypes based on combined mRNA expression and DNA profiling, namely: luminal/androgen receptor (LAR), mesenchymal (MES), basal-like immunosuppressed (BLIS), and basal-like immune activated (BLIA), each with distinct molecular features related to immune regulation and DNA damage response pathways. More recently, transcriptomics data also provided classification into four subtypes based on the cohort of 465 primary TNBCs, i.e., basal-like immune-suppressed, luminal androgen receptor (LAR), immunomodulatory, and mesenchymal-like [10]. Noteworthy, quadruple-negative breast cancer (QNBC) was also distinguished, and it lacks the expression of four receptors such as ER, PR, HER2, and AR. However, this category is not established as a clinical category, and it is not used in clinical practice [31,32].

**Figure 2:**
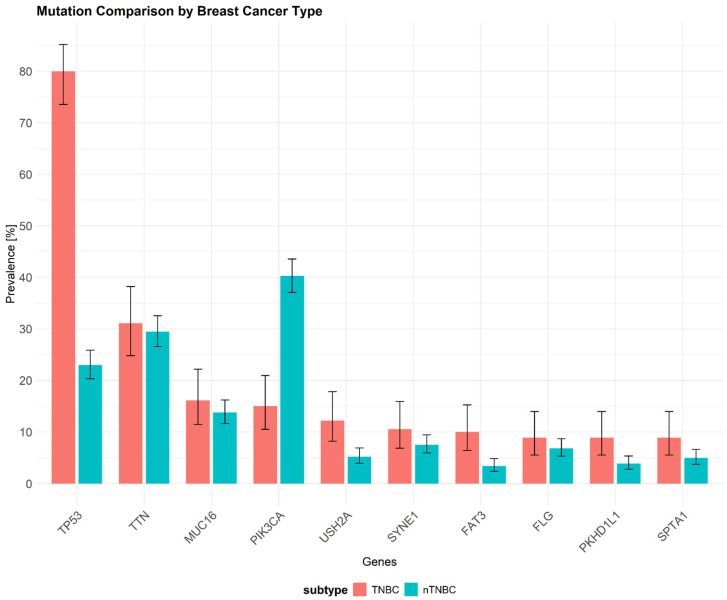
The frequency of somatic mutations in triple-negative breast cancer (TNBC; red) versus other breast cancer subtypes (nTNBC; teal) based on data from The Cancer Genome Atlas (TCGA) database (URL: https://www.cancer.gov/ccg/research/genome-sequencing/tcga (accessed on 16 November 2024)) using TCGAbiolinks package. Mutation data for breast cancer subtypes were processed with the maftools package, and the mutation dataset originates from the study [40] (URL: https://doi.org/10.1016/j.cels.2018.03.002 (accessed on 16 November 2024)). The most frequently mutated genes are shown along with their mutation rates in TNBC samples. The *x*-axis represents the different mutated genes, while the *y*-axis indicates the percentage of samples exhibiting mutations in each gene. *TP53* exhibits the highest mutation frequency (80%) in TNBC, whereas *PIK3CA* shows a notably lower mutation rate compared to other breast cancer subtypes (15%). Data represent ten selected genes with the highest mutation frequency. *TP53*—Tumor Protein P53, *TTN*—Titin, *MUC16*—Mucin 16, *PIK3CA*—Phosphatidylinositol-4,5-Bisphosphate 3-Kinase Catalytic Subunit Alpha, *USH2A*—Usher Syndrome Type 2A, *SYNE1*—Spectrin Repeat Containing Nuclear Envelope Protein 1, *FAT3*—FAT Atypical Cadherin 3, *FLG*—Filaggrin, *PKHD1L1*—Polycystic Kidney and Hepatic disease 1 like 1, *SPTA1*—Spectrin Alpha Chain, Erythrocytic 1.

TNBC is the most commonly observed subtype in patients with *BRCA1* germline mutations [33]. Over 85% of BCs arising in individuals with *BRCA1* pathogenic variants exhibit a triple-negative phenotype. Additionally, about 11% to 19% of patients diagnosed with TNBC have either germline or somatic *BRCA1* mutations, highlighting the genetic link between *BRCA1* alterations and this aggressive breast cancer subtype [34,35]. One of the most prevalent somatic mutations in TNBC are mutations in the *TP53* gene (~80%), which encodes the p53 protein (Figure 2). The p53 protein plays an important role in maintaining homeostasis and genome integrity by regulating cell cycle arrest, DNA repair, and apoptosis [36,37]. Similar to our representation of somatic mutation prevalence from the TCGA database, previous research has shown that mutations in the *TP53* gene occur in over 65–80% of TNBC cases [38,39]. Below, we present recent data on the mutational landscape of TNBC with ten selected genes with the highest mutation frequency (somatic mutations) generated from the TCGA-BRCA database. Notably, *TP53* shows a significantly higher mutation frequency in TNBC (80%) compared to other breast cancer (BC) subtypes (23%), while *PIK3CA* exhibits a lower mutation frequency in TNBC (15%) compared to nTNBC (40.3%). The genetic landscape underlying TNBC is linked to various hallmarks of tumor biology, cell invasion, drug resistance, and immune evasion.

## 3. The Role of Epithelial–Mesenchymal Transition (EMT) in TNBC

### 3.1. The EMT Process Overview

The EMT is a well-conserved, multi-step, reversible process that plays roles in both physiological and pathological contexts, including embryonic development, wound repair, tissue fibrosis, or cancer progression [24,41,42,43]. Complex molecular mechanisms and signaling pathways involved in the EMT are stimulated by the signals from the local microenvironment, such as cytokines, growth factors, hypoxic conditions, and components of the extracellular matrix, initiating the process [44]. The EMT is also driven by the activation of specific transcription factors (TFs) that modify gene expression, resulting in reduced cell–cell adhesion. Cells lose intercellular adhesion and apico-basal polarity with a transition to anterior–posterior axial polarity. These alter the cytoskeletal structure and transition their phenotype from an epithelial to a mesenchymal, affecting both their form and function. Cells simultaneously acquire stem-cell-like traits and migratory and invasive characteristics [45,46]. Dysregulation of EMT is a key factor contributing to cancer-related mortality. It plays a crucial role in metastasis and the development of treatment resistance [47,48].

As aforementioned, EMT is regulated by a number of TFs, such as Snail, Slug, Twist, and ZEB1/2, that inhibit the expression of genes responsible for epithelial properties, while activating genes associated with the mesenchymal phenotype [49]. Moreover, the process is regulated by epigenetic and post-translational modifications (PTMs), including DNA methylation, chromatin remodeling, acetylation of histones and phosphorylation, as well as modifications of non-coding RNAs (ncRNAs), including microRNAs (miRNAs), long non-coding RNAs (lncRNAs), and circular RNAs (circRNAs) [50,51]. Both internal and external factors that influence EMT are presented in Table 1, with internal being intrinsic to the cell and external involving signals and environmental conditions outside the cell.

The reverse process, termed mesenchymal–epithelial transition (MET), involves mesenchymal cells reorganizing their cytoskeleton, restoring apical–basal polarity, and increasing their intercellular adhesion. These changes lead to the formation of an organized epithelial structure [71]. MET facilitates metastatic colonization by enabling cancer cells to re-acquire epithelial traits, such as cell adhesion and proliferation, which are diminished during EMT. While EMT enhances motility, it often suppresses proliferation, requiring tumor cells to alternate between migratory and proliferative states for efficient metastasis. For instance, suppressing Twist1 at secondary sites promotes proliferation and metastatic growth. However, colonization, the rate-limiting step of metastasis, depends on additional molecular events beyond EMT reversal [72]. The underlying mechanisms behind these processes remain incompletely understood. During cancer progression, epithelial-originating cancer cells undergo a process called partial EMT, where they display a combination of mesenchymal and epithelial characteristics, resulting in a hybrid E/M phenotype. This partial EMT is believed to increase the invasiveness of cancer cells, contribute to the formation of circulating tumor cells (CTCs; the cells that have dissociated from the primary tumor, entered the bloodstream, and can migrate throughout the body) and cancer stem cells (CSCs), and foster resistance to anti-cancer therapies [73]. Interestingly, CTCs from BC patients showed significant enrichment of mesenchymal markers compared to primary tumor cells, supporting the role of EMT in metastasis. Serial CTC monitoring revealed that mesenchymal CTCs were associated with disease progression, with dynamic shifts in cell fates observed during therapy cycles. These findings highlight the involvement of EMT regulators, e.g., TGF-β (transforming growth factor-beta), in the spread of BC [74].

### 3.2. EMT Protein Markers in TNBC and Their Role in Tumor Progression

The EMT process in TNBC involves the expression of specific protein markers that define and regulate the shift from an epithelial to a mesenchymal phenotype. These markers are categorized into epithelial markers, such as E-cadherin, cytokeratins, zonula occludens-1, claudin, occludin, and mesenchymal markers, including N-cadherin, vimentin, and fibronectin, alongside other EMT-related proteins like MMPs (matrix metalloproteinases). The process of EMT involves cytoskeletal reorganization, the loss of epithelial features, such as apical–basal polarity and strong cell–cell adhesion, coupled with the acquisition of mesenchymal traits, including front–back polarity and invasive morphology, the loss of expression of adhesion proteins such as E-cadherin, and increased expression of mesenchymal markers such as N-cadherin, vimentin and fibronectin [75].

Some of the proteins, however, cannot be classified as strictly epithelial or mesenchymal markers, as their roles and expression patterns are highly context-dependent. For instance, integrins, belonging to the family of transmembrane receptors that mediate cell adhesion to the ECM [76], may be associated either with epithelial or mesenchymal phenotype. Integrin α6β4 is often associated with epithelial cells, where it is predominantly expressed. Its expression tends to decrease as cells lose their epithelial characteristics [77]. It also shows a high expression level in TNBC, where it plays a significant role in tumor progression and drug resistance [78]. On the other hand, integrins like, e.g., α5β1, αvβ6, and αvβ3 are more commonly upregulated in mesenchymal cells. These integrins interact with components of the ECM, such as fibronectin and vitronectin, promoting migration, invasion, and survival, hallmarks of mesenchymal phenotypes during EMT [79]. Other proteins important in the context of TNBC progression, migration, invasion, and treatment resistance that are also linked to the EMT process are integrin α7 protein encoded by the *ITGA7* gene and semaphorin 6 D encoded by the *SEMA6D* gene [80,81].

**Figure 3:**
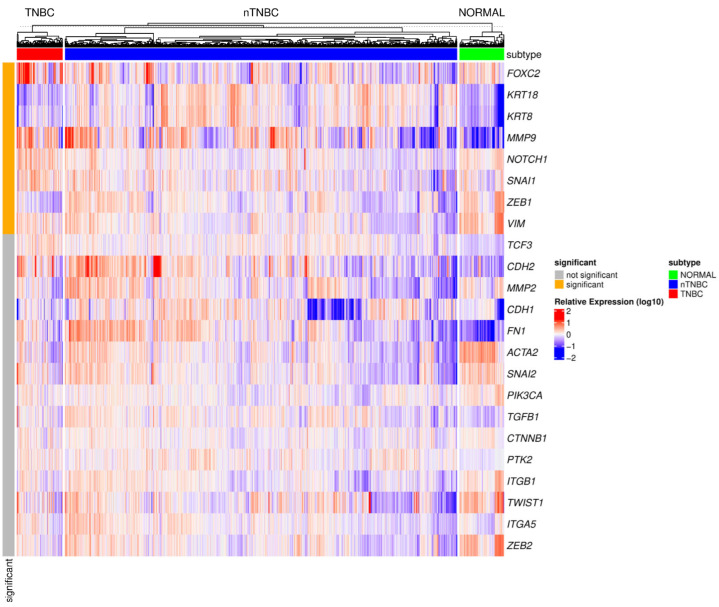
A heatmap representing the gene expression profiles of EMT-related genes across TNBC, other breast cancer subtypes (nTNBC), and normal tissues. The data were derived from the TCGA database, with expression levels normalized, log-transformed, and standardized for comparability. The analysis incorporates mutation data from the Broad Institute TCGA Genome Data Analysis Center (2016), generated using MutSig 2CV v3.1 [82]. Genes with an expression threshold of |log_2_(expression)| ≥ log_2_(1.5) and statistical significance adjusted *p*-value < 0.05 were included (significant—orange and not significant—gray). Hierarchical clustering was performed for samples, revealing distinct expression patterns that differentiate TNBC from nTNBC. Expression levels above the median are shown in red (upregulated), those below the median in blue (downregulated), and values close to the median in white. Color intensity reflects the magnitude of deviation from the median. *FOXC2*—Forkhead Box C2, *KRT18*—Keratin 18, *KRT8*—Keratin 8, *MMP9*—Matrix Metallopeptidase 9, *NOTCH1*—Notch Receptor 1, *SNAI1*—Snail Family Transcriptional Repressor 1, *ZEB1*—Zinc Finger E-box Binding Homeobox 1, *VIM*—Vimentin, *TCF3*—Transcription Factor 3, *CDH2*—Cadherin 2, *MMP2*—Matrix Metallopeptidase 2, *CDH1*—Cadherin 1, *FN1*—Fibronectin 1, *ACTA2*—Actin Alpha Cardiac Muscle 2, *SNAI2*—Snail Family Transcriptional Repressor 2, *PIK3CA*—Phosphatidylinositol 4,5-bisphosphate 3-kinase Catalytic Subunit Alpha, *TGFB1*—Transforming Growth Factor Beta 1, *CTNNB1*—Catenin Beta 1, *PTK2*—Protein Tyrosine Kinase 2, *ITGB1*—Integrin Subunit Beta 1, *TWIST1*—Twist Family BHLH Transcription Factor 1, *ITGA5*—Integrin Subunit Alpha 5, *ZEB2*—Zinc Finger E-box Binding Homeobox 2.

To evaluate the expression of genes encoding some of the protein markers involved in EMT in TNBC, as well as other EMT-related genes, we generated a heatmap that represents the gene expression profiles across three phenotypic groups: TNBC, other breast cancer subtypes (nTNBC), and normal tissues (Figure 3). Distinct expression patterns that differentiate TNBC from nTNBC were assessed. Gene expression data were derived from the TCGA-BRCA dataset, based on RNA-seq analysis, with samples grouped according to their phenotypic classification. Expression levels for each gene were normalized, log-transformed, and standardized to ensure comparability across samples. The threshold was set as |log_2_(expression)| ≥ log_2_(1.5), and statistical significance was considered as adjusted *p*-value < 0.05. The heatmap was constructed using hierarchical clustering of samples, enabling the identification of expression patterns and clustering trends that distinguish TNBC from other breast cancer subtypes.

This analysis revealed a significant upregulation of genes associated with EMT in TNBC compared to nTNBC. Specifically, increased expression was observed for EMT transcription factors (e.g., *SNAIL1*, *ZEB1*), transcriptional regulators (e.g., *FOXC2*, *NOTCH1*), and EMT markers (e.g., *VIM*, *KRT18*, *KRT8*) as well as *MMP9*. Hierarchical clustering and heatmap analysis highlighted these distinct molecular profiles separating TNBC from other BC subtypes (nTNBC) in the context of EMT-related genes.

#### 3.2.1. The Main Epithelial Markers and Their Involvement in TNBC Progression

There are several proteins associated with epithelial cell phenotypes that are frequently used to characterize the epithelial features of cancer cells and also play an important role in TNBC progression. Among the most common epithelial markers that exhibit decreased expression in TNBC are E-cadherin, β-catenin, and cytokeratins. Additionally, proteins integral to tight junctions (TJs), such as claudins and occludin, as well as scaffolding proteins like ZO-1 (zonula occludens-1), are linked to the epithelial state and often show reduced expression in TNBC, reflecting a loss of epithelial characteristics during disease progression. The major epithelial markers and proteins associated with EMT that are involved in TNBC progression are described below.

##### E-Cadherin

E-cadherin, encoded by the *CDH1* gene, is a key adhesion protein that plays a role in maintaining epithelial integrity by forming adherens junctions, which are essential for tight intercellular adhesion. Under physiological conditions, E-cadherin acts as a suppressor of tumor invasion [83,84]. As aforementioned, the process of EMT plays a pivotal role in tumor cell invasion and metastasis, enabling cancer cells to transition from an epithelial to a mesenchymal phenotype, thus enhancing their motility and capacity to overcome tissue barriers. E-cadherin loss or reduced expression is one of the key markers of EMT, observed in approximately 67% of TNBC patients [85]. The reduction or entire loss of E-cadherin in TNBC facilitates tumor progression by promoting the EMT process [86]. Tang et al. [85] have shown that the loss of E-cadherin is significantly correlated with poorer clinical outcomes for TNBC patients, including shorter DFS and OS. TNBC patients with reduced E-cadherin expression exhibited significantly shorter DFS (*p* = 0.016) and OS (*p* = 0.012) compared to those with higher E-cadherin expression. This loss of E-cadherin expression is also associated with poor prognostic features, such as, for instance, tumor size and lymph node status, further highlighting the role of E-cadherin in TNBC progression [85]. Kashiwagi et al. [87] observed that among a cohort of 574 primary breast cancer patients, reduced E-cadherin expression was more frequent in TNBC and correlated with worse patients’ outcomes, particularly in clinical stage II of the disease. Multivariate analysis confirmed reduced E-cadherin expression as an independent prognostic factor, suggesting its potential as a marker for defining TNBC subgroups and guiding treatment decisions [87]. A study by Jeong et al. [88] found that EMT characteristics were particularly prominent in TNBC compared to HR-positive breast cancer. These features were significantly associated with high histological grade (*p*-value < 0.001) as assessed by immunohistochemistry (IHC). The study also observed a decreased expression of E-cadherin in 16.7% of TNBC patients, indicating a loss of epithelial markers associated with the transition to a mesenchymal phenotype in TNBC. The presence of mesenchymal markers, such as vimentin (24.5% of patients) and N-cadherin (13.7% of patients) [88] suggests that EMT plays a crucial role in the aggressive nature of this tumor subtype, potentially contributing to poorer prognosis and increased metastatic potential.

##### β-Catenin

β-catenin serves two main roles. First, it is an essential part of E-cadherin and other cadherin-based cell adhesion complexes, which are important for maintaining the structural integrity and functional organization of epithelial cells and tissues. Second, β-catenin acts as a primary mediator in the canonical Wnt (Wingless/Integrated) signaling pathway. When this pathway is activated, β-catenin accumulates in the cytoplasm and subsequently translocates to the nucleus, where it interacts with T-cell factor (TCF)/lymphoid enhancer factor (LEF) transcription factors to regulate the expression of Wnt target genes [89,90]. In this way, β-catenin is central to both cadherin-mediated cell adhesion and the Wnt signaling. It has been shown that loss of cadherin-mediated adhesion promotes β-catenin release, leading to increased signaling activity, a process facilitated by proteases, protein kinases, and other molecular factors. Moreover, the Wnt signaling pathway is interconnected with cadherin adhesion as it targets related genes, including transcription factor-encoding genes (e.g, TWIST1, SLUG1), genes encoding metalloproteases and/or the cell adhesion molecule L1; both of the latter promote E-cadherin degradation. These interconnections provide a mechanism by which the loss of cadherin and enhanced Wnt signaling drive EMT, contributing to both TNBC development and progression [91]. In TNBC, a frequently activated Wnt signaling pathway contributes to the aggressive nature and poor prognosis of this subtype, regulating key tumorigenic behaviors, such as cell migration, stemness, anchorage-independent growth, and chemoresistance. Given its central role in TNBC progression, targeting β-catenin offers potential therapeutic value in managing this challenging cancer subtype [92]. In a study of 72 TNBC cases, reduced membranous expression of E-cadherin and β-catenin was observed in 56% of tumors, with a strong correlation between these two markers. The reduced expression level of both was significantly associated with poor OS and disease-specific survival (DSS), and multivariate analysis confirmed that β-catenin expression was an independent prognostic factor. These findings highlight the important role of E-cadherin and β-catenin as prognostic markers in TNBC, independent of conventional clinicopathologic factors [93]. Buechel et al. [94] investigated the distinct roles of β-catenin’s cell adhesion and transcriptional functions in metastatic breast cancer using the mouse mammary tumor virus–polyoma middle T antigen (MMTV-PyMT) model. They found that both functions are crucial for tumor cell survival, with β-catenin’s transcriptional activity being essential for EMT and metastasis. The loss of β-catenin’s transcriptional activity resulted in reduced primary tumor growth, invasion, and metastasis, underlining its critical role in cancer progression. This study highlights the importance of β-catenin’s transcriptional function in driving malignant BC progression [94].

##### Cytokeratins

Cytokeratins (CKs), as key components of the cytoskeleton in epithelial cells, serve as markers of epithelial differentiation, particularly in the context of TNBC [95,96]. CKs such as CK5/6, CK14, and CK8/18 are markers of basal-like and luminal epithelial differentiation [97,98], and their expression patterns reflect the heterogeneity of the TNBC subtype. It has been shown that the expression of CK5/6 and CK14 in TNBC correlates with increased tumor aggressiveness and poorer patients’ survival and prognosis [95,99,100]. In a recent study examining EMT in breast cancer subtypes across different species, human TNBC showed decreased expression of CK8/18 and increased expression of CK5/6 and CK14 as assessed by IHC on eleven specimens. These changes contribute to the enhanced migratory and invasive capabilities of TNBC cells, thereby promoting disease progression and metastasis [101]. However, larger cohorts should be analyzed using IHC or complementary methodologies to validate the expression levels of these proteins, their clinical significance, and implications. CKs may potentially also serve as therapeutic targets in TNBC.

##### Claudins

Claudins (CLDNs) are essential cell adhesion molecules that form TJs, regulate paracellular permeability, and maintain cell polarity [102,103]. Disruption of TJs can trigger EMT, which leads to the loss of cell–cell contacts, including the loss of E-cadherin, facilitating tumor invasion and metastasis [42,104,105]. Genomic studies have uncovered a claudin-low subtype of triple-negative breast cancer (TNBC), characterized by reduced expression of CLDN3 (claudin 3), CLDN4 (claudin 4), CLDN7 (claudin 7), and E-cadherin [106,107]. More recently, Geoffroy et al. [108] have shown that the loss of *CLDN1* (claudin 1) correlates with increased invasiveness and higher recurrence of TNBC. Overall, the expression of CLDN1 is absent or strongly diminished in 76% of TNBC cases [109]. Transient overexpression of CLDN1 in MDA-MB-231 and Hs578T TNBC cell lines (which typically also have low CLDN1 expression), reduced cell migration, as demonstrated by wound healing and transwell assays. Stable overexpression of CLDN1 in MDA-MB-231 clones correlated with reduced cell migration, enhanced cell–cell aggregation, and the formation of β-catenin adherens junctions and occludin TJs. CLDN1 was also identified as a key factor in inhibiting cell migration, linked to the loss of stress fibers [108]. In patients with TNBC, altered expression of CLDNs has been associated with aggressive disease characteristics and poor patients’ prognosis. Specifically, high expression levels of CLDN1, CLDN4, and CLDN7 in TNBCs were more frequently observed compared to other BC subtypes, correlating with increased proliferative activity as indicated by the Ki67 labeling index. Notably, the combination of high CLDN4 and low CLDN7 expression was found to be an independent predictor of axillary nodal metastasis. Furthermore, low expression of CLDN1 and CLDN7, along with the loss of E-cadherin, was associated with poor recurrence-free survival (RFS), highlighting their potential as prognostic markers in TNBC [110]. These findings emphasize the involvement of claudins in EMT, metastasis, and overall tumor aggressiveness in patients with TNBC.

#### 3.2.2. The Main Mesenchymal Markers in TNBC

Induction of EMT in TNBC leads to an increase in the expression of mesenchymal markers, including vimentin, N-cadherin, fibronectin, and other proteins related to mesenchymal states such as Fsp1 (fibroblasts specific protein 1) or MMPs (matrix metalloproteinases) that are activated along with pro-migratory signaling [111].

##### Vimentin

In the physiological context, vimentin (VIM) plays a significant role in several cellular functions, including maintaining cell shape and anchoring organelles. It is a key regulator of the cytoskeleton, supporting not only dynamic changes in cell shape but also enabling cell migration and invasion. By reorganizing the cytoskeleton, vimentin also facilitates the detachment of cancer cells from the primary tumor and their penetration into surrounding tissues. Moreover, it aids in the reorganization of actin filaments, further enhancing the motility of cancer cells [112,113,114].

Vimentin, as a mesenchymal marker in TNBC, is highly expressed in this BC subtype compared with other subtypes [115] and correlates with cancer invasiveness. Vimentin expression in TNBC is regulated by EMT-associated transcription factors, e.g., Snail1 and Twist1, which downregulate epithelial markers like E-cadherin, while promoting the expression of mesenchymal markers like vimentin or N-cadherin [49,116]. Ki-67 LI expression in TNBC cases is also significantly correlated with vimentin expression, highlighting its association with the EMT process [4]. TNBC cells (MDA-MB-231, SUM159-PT cell lines) with high vimentin expression exhibit enhanced migratory and invasive capabilities as observed in vitro and in a zebrafish model. This contributes to increased aggressiveness, which is associated with chemotherapeutic treatment-induced changes, where vimentin contributes to increased sphere formation and AKT phosphorylation [117]. These findings highlight vimentin as an important player in promoting invasion and potential drug resistance in TNBC, pointing to its potential as a therapeutic target in combating TNBC aggressiveness and recurrence. IHC-based studies conducted by Jeong et al. [88] revealed that approximately 24.5% of TNBC patients show high vimentin expression, compared to only 4.1% in receptor-positive cancers. This also strongly suggests that vimentin is closely associated with more aggressive forms of breast cancer.

Recently, Grasset et al. [118] showed that TNBC often displays EMT markers, though highlighting that the role of EMT in TNBC metastasis remains unclear. Through genetically engineered mouse models (GEMMs), patient-derived xenografts (PDXs), and patient samples, they identified large populations of hybrid epithelial/mesenchymal (E/M) cells leading to cancer invasion. The mesenchymal marker vimentin was found to enhance this invasion but limit metastatic growth. TNBC employs a vimentin-driven hybrid EMT, facilitating the formation of metastases with a mix of epithelial and mesenchymal traits. The decrease in vimentin expression level significantly decreased invasion capacity in the 3D culture models (both GEMM and PDX). EMT TFs showed distinct roles in invasion versus colony formation, with single-cell RNA sequencing (scRNA-seq) analysis revealing three EMT-related patterns. Notably, TNBC cells shifted from epithelial to hybrid E/M and mesenchymal patterns during invasion, and largely from epithelial to hybrid E/M state during colony formation. Both GEMM and patient samples displayed significant variability within and between metastases, suggesting multiple molecular strategies for organ colonization [118]. These results highlight the complex role of EMT in TNBC metastasis.

##### N-Cadherin

N-cadherin, encoded by the *CDH2* gene, is a calcium-dependent adhesion molecule, characteristic of mesenchymal cells. During EMT, the downregulation of E-cadherin expression contributes to the loss of adhesive properties in epithelial cells and the acquisition of mesenchymal traits, marked by the upregulation of N-cadherin. This so-called “cadherin switch” is considered a hallmark of EMT, also significantly enhancing the migratory potential and cancer invasiveness and deteriorating patients’ outcomes [75,119,120]. The expression of N-cadherin is regulated by several EMT-associated transcription factors, such as Snail, Slug, and Twist, which repress epithelial markers while activating mesenchymal markers [66,121]. Immunohistochemical analyses revealed that N-cadherin is expressed in approximately 13.7% of TNBC patients, compared to only 5.9% in hormone HR-positive breast cancers. This increased expression in TNBC correlates with a higher histological grade and a more aggressive disease phenotype. Furthermore, N-cadherin expression is associated with shorter DFS and OS [88]. N-cadherin has also been shown to significantly impact prognosis, as its expression is linked to poorer outcomes in various cancer types. In line with this, Aleskandarany et al. [122] further emphasized the role of N-cadherin as a marker of aggressive disease, strengthening its potential as a prognostic factor in TNBC. Furthermore, N-cadherin facilitates the interaction between cancer cells and the stromal environment, particularly with cancer-associated fibroblasts (CAFs), by mediating a heterophilic adhesion that promotes invasion. High N-cadherin expression also enhances the mechanical activity of this interaction, supporting cancer cell invasion and repolarization of CAFs, which is crucial for the progression of cancer and potential metastasis [123]. These findings highlight the importance of N-cadherin in tumor progression, as its role in regulating stromal interactions contributes to both cancer cell spread and the dynamic reorganization of the TME. The expression of N-cadherin is also associated with plasticity and hybrid epithelial/mesenchymal (E/M) phenotypes in TNBC cells. These phenotypes increase cell plasticity, enabling adaptation to variable microenvironmental conditions during metastasis and contributing to therapeutic resistance [118,124].

Targeting N-cadherin or its regulatory pathways could provide a therapeutic strategy for TNBC. Blocking N-cadherin-mediated intercellular interactions through peptides or small molecules could offer a novel approach to treating this cancer subtype [125]. Early investigations into N-cadherin antagonists, such as ADH-1 (adherex 1), have demonstrated their potential as anti-cancer agents, with promising results in clinical trials for melanoma [126] and indications of efficacy in ovarian cancer [127]. In contrast, our earlier studies demonstrated that histone deacetylase inhibitors (HDIs), such as valproic acid and vorinostat, inhibit the proliferation and migratory activity of TNBC cell lines, while unexpectedly increasing the expression of N-cadherin in these cells. We hypothesized that cancer cells might adapt to HDIs’ treatment by upregulating structural proteins like N-cadherin to enhance their survival. Alternatively, the epigenetic mechanisms of HDIs might influence cadherin gene expression as “a side effect” of their effects on chromatin structure and activity. This phenomenon is of clinical interest, as understanding the phenotypic changes in tumor cells could be crucial for optimizing patients’ therapy [128]. Further research is necessary to explore the full therapeutic potential of these antagonists, small-molecule or histone deacetylase inhibitors, and monoclonal antibodies targeting N-cadherin in the EMT process and associated pathways in TNBC.

## 4. Molecular Mechanisms Related to EMT in TNBC Progression and Their Implications for Drug Resistance

EMT has gained significant attention as an emerging mechanism contributing to cancer drug resistance [129,130,131,132]. Cancer cells with mesenchymal characteristics frequently exhibit inherent resistance [133]. EMT plays a crucial role in promoting such resistance in various cancer types, including TNBC [61,131]. This is completed via several key mechanisms. As aforementioned, during EMT, cancer cells lose their epithelial characteristics, such as cell–cell adhesion and polarity, and acquire mesenchymal features that enhance their migratory and invasive abilities. This transformation is driven by the activation of several TFs, such as Snail, Slug, Twist, and Zeb1, which regulate the expression of mesenchymal markers such as vimentin and N-cadherin [134]. Such molecular changes facilitate not only increased cell motility but also promote pro-survival signaling pathways that contribute to treatment resistance [23]. The EMT process can influence the effectiveness of TNBC therapies by enhancing the survival of cancer cells in the heterogeneous TME [135]. The relation between EMT and signaling pathways is reciprocal. The EMT process can not only modulate particular signaling pathways but very often signaling pathways are well-established drivers of EMT, contributing to chemoresistance.

### 4.1. Signaling Pathways Associated with EMT in TNBC

The development of cancer, including TNBC, is complex and involves a series of genetic changes, activation of specific transcription factors over time, and alterations of various signaling pathways that may interact with other cellular pathways. Under physiological conditions, intricate signal transduction networks, such as, e.g., the transforming growth factor-beta (TGF-β) pathway, are critical in maintaining cellular balance. Here, we list the main signaling pathways related to EMT in TNBC (Table 2) that are associated with this BC subtype progression and chemoresistance. We also briefly discuss the role of the selected pathways in the context of EMT in TNBC with their involvement in resistance to anticancer agents.

#### 4.1.1. TGF-β Signaling in the Context of EMT and TNBC

TGF-β (transforming growth factor-beta) is a family of closely related cytokine proteins that play a dual role in cancer: initially acting as a tumor suppressor in early disease stages but later promoting tumor growth by driving EMT, which contributes to metastasis and resistance to chemotherapy in advanced cancer stages [149,150,151]. TGF-β signaling plays a crucial role in activating EMT in BC, including TNBC [152].

TGF-β induces both SMAD2/3-dependent and non-SMAD (Sma and Mad) signaling to promote EMT. In SMAD signaling, TGF-β binds to receptors, activating the SMAD2/3/4 complex. Non-SMAD pathways involve the PI3K/AKT, Ras/Raf/MEK/ERK, and Wnt/β-catenin pathways, all contributing to the induction of mesenchymal traits [153,154]. TGF-β also activates Ras/Raf/MEK/ERK through the ShcA (Src homology 2 domain-containing transforming protein A) and GRB2 (growth factor receptor-bound protein 2) proteins [155]. Furthermore, TGF-β signaling induces RhoA degradation, destabilizing TJs and promoting EMT. The PAR6 (partitioning defective 6) protein, involved in cell polarity, is crucial in TGF-β-driven EMT, and blocking PAR6 signaling suppresses EMT and metastasis [156]. TGF-β also regulates cytoskeletal dynamics through RhoA and CDC42, influencing cell migration [157].

Additionally, TGF-β interacts with the PI3K/AKT and Wnt/β-catenin pathways to further promote EMT and cancer cell migration [158,159]. Studies show that β-catenin and TGF-β signaling cooperate to induce a mesenchymal phenotype, with inhibition of both pathways leading to the reversion of cells to an epithelial state [160]. Recent studies have suggested that SDC1 (a cell surface proteoglycan syndecan-1) controls the migration of MDA-MB-231 TNBC cells via a mechanism dependent on TGF-β1-SMAD signaling and E-cadherin [161], while a transcription factor RUNX2 (runt-related transcription factor 2) influences chemoresistance by modulating cancer cell stemness through its direct interaction with TGF-β [162]. Prolonged TGF-β exposure has been linked to resistance to anti-cancer drugs such as doxorubicin (DOX), cisplatin, or cyclophosphamide in human mammary epithelial cells (HMLER cell line modified to exhibit EMT features) [163]. This signaling pathway has also been shown to be a key regulator of epirubicin resistance in TNBC, influencing both EMT and cell death mechanisms [61].

In TNBC, the TGF-β-induced EMT pathway significantly contributes to chemoresistance, as the transition to a mesenchymal state [86] is associated with potentially reduced drug sensitivity and enhanced survival of cancer cells. Hence, targeting TGF-β signaling, either through inhibition of its receptors or downstream effectors is a potential therapeutic strategy to overcome chemoresistance and improve patients’ outcomes in TNBC. Recently, it has also been shown that TGF-β-induced EMT in TNBC and subsequently tumor progression and resistance to anticancer drugs is mediated by aurora-A kinase. The so-called breast tumor-initiating cells (BTICs) undergoing EMT exhibit high ALDH1 (aldehyde dehydrogenase 1) activity resulting in the suppression of chemotherapy-induced apoptosis. Aurora-A plays a crucial role in promoting the expression of the *SNAI1* gene in response to TGF-β signaling. This, in turn, leads to an increase in the population of ALDH1-high BTICs, enhances their ability to self-renew, and contributes to their resistance to chemotherapy in TNBC experimental models, using MDA-MB-231 TNBC cell line or xenografts mouse models utilizing MDA-MB- 231 and TNBC-M40 cells [137].

#### 4.1.2. NF-κB Signaling in the Context of EMT and TNBC

NF-κB (nuclear factor-kappaB) is among the most widely recognized molecular pathways involved in cancer, playing an oncogenic role [164,165]. It can transduce its signaling via canonical and non-canonical pathways. The canonical pathway is stimulated with, e.g., IL-1β (interleukin-1β) and tumor necrosis factor (TNF), leading to translocation of NF-κB to the nucleus, which in turn impacts gene expression levels [138,166]. This signaling pathway also plays a key role in TNBC chemoresistance [167] by promoting cell survival, enhancing their ability to evade apoptosis, and regulating the EMT process by inducing transcription of several genes involved in the EMT initiation. Various EMT-associated genes, including *SLUG*, *SIP1*, *TWIST1*, and *MMP11*, have also been suggested to be regulated by NF-κB signaling in BC [168].

In TNBC, the protein ADAM9, a member of a disintegrin and metalloproteinases (ADAMs) family, whose members are involved in the EMT process in various cancers, has been shown to be upregulated in TNBC in both cell lines and patients’ samples. It has been reported that the overexpression of ADAM9 promotes proliferation, migration, and invasion in TNBC by activating the AKT/NF-κB pathway, suggesting that this protein may be a potential therapeutic target for TNBC [169]. Recently, it has also been shown that the NEDD9 (neural precursor cell expressed, developmentally downregulated 9) protein plays a role in BC progression and metastasis. Analysis of the CPTAC (Clinical Proteomic Tumor Analysis Consortium) database revealed that NEDD9 is upregulated in BC tissues and associated with poor prognosis. Functional experiments demonstrated that NEDD9 overexpression promotes BC growth (including TNBC; MDA-MB-231 cell line and mouse models) and metastasis, disrupts mammary epithelial acinus formation, and triggers EMT. These effects are reversed by silencing NEDD9. Mechanistically, NEDD9 enhances its expression by inhibiting HDAC4 (histone deacetylase 4) activity, leading to increased H3K9 acetylation of the NEDD9 promoter and activation of the FAK (focal adhesion kinase)/NF-κB signaling pathway. Moreover, NEDD9 drives IL-6 (interleukin 6) secretion, fostering anti-inflammatory macrophage polarization in the TME and enhancing BC cell invasiveness [170].

Moreover, some recent studies have focused on natural compounds such as curcumin and caffeic acid phenethyl ester (CAPE) to investigate their effects on TNBC in relation to the EMT process. Curcumin has been proposed to prevent EMT by downregulating the expression of proteins linked to the PI3K/AKT/mTOR signaling pathways, thereby reducing the migratory potential of TNBC cells. CAPE was also proposed to have the ability to reduce inflammation and metastasis markers expression levels in TNBC, particularly through its impact on NF-κB signaling activated by tumor necrosis factor-alpha (TNF-α) and DOX [171,172]. However, both studies require further research into detailed molecular mechanisms behind these processes. Additionally, flavonoids such as kaempferol, luteolin, apigenin, and others have been reported to modulate the NF-κB pathway in BC via the inhibition of EMT, regulation of growth factor receptors, and modulation of signaling pathways such as PI3K/AKT and MAPK/ERK. These compounds also influence apoptosis, autophagy, regulation of ATP-binding cassette (ABC) transporters, and CSCs activity, contributing to reduced therapy resistance [173]. Overall, these findings highlight the potential of natural compounds to target EMT and drug resistance mechanisms in TNBC. However, further research is needed to clarify the molecular mechanisms and clinical applicability of these strategies.

#### 4.1.3. Notch Signaling in the Context of EMT and TNBC

Notch, an ancient and highly conserved signaling pathway, plays a role in various biological processes, including organ formation, tissue function, and repair [174]. The Notch signaling pathway has also been identified as a key factor in the development, progression, and drug resistance in TNBC. Notably, this pathway can also interact with TGF-β signaling [175]. Firstly, Notch receptors are involved in regulating the behavior of tumor-initiating cells (TICs) and the pathogenesis of TNBC. Secondly, substantial evidence supports the role of the Notch pathway in the maintenance and expansion of CSCs, a subpopulation of tumor cells associated with drug resistance, metastasis, and tumor recurrence. Notch signaling promotes the survival of CSCs, which are highly resilient to chemotherapy, largely due to their ability to remain quiescent and evade drug-induced apoptosis. Lastly, the expression and activation of Notch receptors (Notch1, Notch2, Notch3, and Notch4) are closely associated with aggressive clinical and biological features of BC, including invasiveness and chemoresistance [176,177].

The activation of Notch receptors, particularly Notch1, upregulates TFs such as Snail, Slug, and Twist, which are critical drivers of EMT. This activation promotes cancer cell migration and invasion by modifying cell adhesion [178]. It has also been shown that overexpression of Notch1 correlates with higher invasiveness of BC cells and their ability to metastasize. On the other hand, inhibition of the Notch pathway leads to the reversal of EMT, which results in reduced migration and invasion of cancer cells [179]. In addition to promoting EMT, Notch signaling contributes to chemoresistance in TNBC. Notch1 activation induces the ATR-CHK1 (ataxia–telangiectasia and Rad3-related kinase-checkpoint kinase 1) signaling cascade, which restores cell cycle checkpoints and prevents mitotic catastrophe, making the cells resistant to chemotherapy [180]. Similarly to TGF-β, targeting this pathway offers a potential therapeutic strategy to prevent metastasis and improve patients’ outcomes. However, given its complexity, further research is needed to develop effective therapies for this aggressive BC subtype.

#### 4.1.4. Wnt Signaling in the Context of EMT and TNBC

Another evolutionary conserved cellular pathway that has been linked to cancer, including TNBC, is the Wnt signaling pathway, which is normally implicated in the development and many other cellular functions [181]. Aberrations in Wnt signaling are linked to tumor progression, invasion, and the development of chemoresistance [182,183]. Wnt ligands are a group of nineteen secreted proteins that trigger signaling by binding to Frizzled receptors and LRP5/6 (low-density lipoprotein receptor-related protein 5 and 6) co-receptors. This signaling can occur either via the canonical pathway involving β-catenin or through various other proteins in a noncanonical way (recently reviewed in [184]).

In TNBC, the Wnt pathway interacts with several growth factors and other signaling networks, with β-catenin acting as a crucial downstream mediator. Key receptor tyrosine kinases (RTKs) central to these signaling processes are EGFR (epidermal growth factor receptor), TGF-β receptor, IGFR (insulin-like growth factor receptor), and VEGFR (vascular endothelial growth factor receptor). The dysregulation of the Wnt/β-catenin pathway may hinder the effectiveness of RTK inhibitors in clinical settings, contributing to the limited success of these treatments in clinical trials [183,185]. Wnt/β-catenin signaling is hyperactivated in TNBC, although the molecular mechanism behind this activation remains largely unknown. Liu et al. [186] have demonstrated that the Wnt/β-catenin pathway is activated by microRNAs (miRNAs)–miR-221 and miR-222. These miRNAs are significantly and specifically upregulated in human TNBC cells (MDA-MB-468, Hs 578T, HCC1937, and MDA-MB-231 cell lines) and negatively correlate with survival rates of TNBC patients. Moreover, they promote the viability, proliferation, EMT process, and migration of TNBC cells. miR-221 and miR-222 activate the Wnt/β-catenin pathway in TNBC cells by targeting multiple negative regulators, leading to increased nuclear β-catenin accumulation and transcriptional activity of Wnt/β-catenin target genes. Furthermore, a positive feedback loop exists where Wnt/β-catenin signaling upregulates miR-221 and miR-222 expression, increasing tumorigenesis [186]. The in vitro studies by Xu et al. [92] showed that β-catenin expression correlates with TNBC chemoresistance as its knockdown made cells more sensitive to chemotherapeutic drugs, such as doxorubicin and cisplatin.

### 4.2. Other Important Mechanisms of EMT-Related Chemoresistance in TNBC

**Figure 4:**
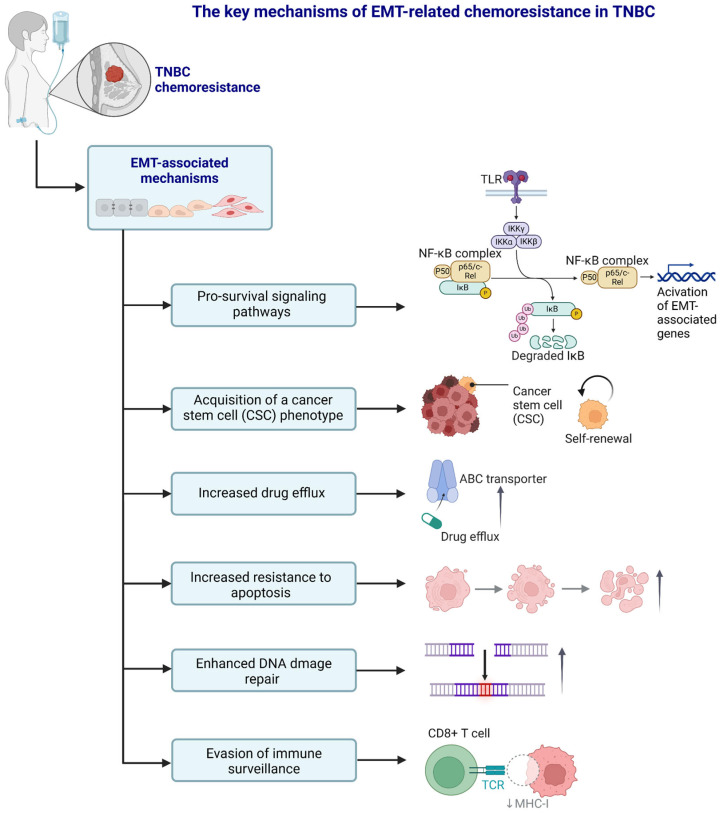
A schematic representation of the selected, key EMT-related chemoresistance mechanisms in TNBC. Created in https://BioRender.com. The upper NF-κB part modified from the BioRender template after Ruslan Medzhitov et al. (Janeway’s Immunobiology, 8th Edition; Garland Science, New York, 2012).

In the previous section, we focused on several key molecular signaling pathways significantly linked to EMT and its contribution to TNBC progression and drug resistance. Below, we describe other mechanisms and factors that contribute to chemoresistance in TNBC and are associated with the EMT process. These include the acquisition of a cancer stem cell (CSC) phenotype, increased drug efflux, increased resistance to apoptosis, enhanced DNA damage repair processes, and cancer cells’ ability to evade immune surveillance (Figure 4).

#### 4.2.1. Acquisition of a Cancer Stem Cell (CSC) Phenotype

It has been shown that during the EMT process, less differentiated cells can be generated that give rise to CSCs [187]. CSCs possess the ability to self-renew and can generate either new CSCs or differentiated daughter cells [188] with different clonal populations, resulting in intratumoral heterogeneity [189,190]. The CSC phenotype is central to the EMT-driven drug resistance in TNBC, and TNBC cells exhibit an enrichment of various stemness pathways compared to other BC subtypes [177]. These, for example, include pathways such as Wnt/β-catenin and Notch [191,192] or some regulators of the EMT process such as Snail and Twist transcription factors [193,194], and other factors such as the TME hypoxia and increased activity of OCT4 (octamer-binding transcription factor 4) and SOX2 (SRY-box transcription factor 2) pluripotency mediators or MYC (master regulator of cell cycle entry and proliferative metabolism) (reviewed in [177]). Since stemness pathways in TNBC are more significantly dysregulated compared to non-TNBC tumors, TNBC cancer stem cells (TNBCSCs) are a particularly challenging clinical phenotype. TNBCSCs are resistant to conventional chemotherapies and possess complex mechanisms for survival and metastasis. These correlate with poor prognosis and resistance to treatment [177,195]. A positive correlation was observed between the expression of stem cell markers (CD44 and ALDH1) and the reduced survival rates in TNBC patients [196,197,198]. Targeting the stemness in TNBC may, therefore, be a promising therapeutic approach. In this context, recent studies have explored natural compounds like tetrandrine, a plant alkaloid, which shows potential in reducing CSCs’ traits and EMT. Tetrandrine has been shown to suppress stem cell markers such as CD44+CD24−/low, inhibit mammosphere formation, and prevent cell migration and invasion. Moreover, tetrandrine regulates EMT-related proteins such as E-cadherin, vimentin, and occludin. These suggest its efficacy in targeting the mechanisms underlying TNBC metastasis and treatment resistance [199]. These findings support tetrandrine as a promising anti-TNBC agent, particularly in targeting TNBCSCs and the EMT process.

DOX, a common chemotherapeutic used in TNBC, has been recently shown to induce EMT in CSCs, making the cancer more aggressive. To overcome this treatment limitation, Sarkar et al. [200] combined DOX with DIM (indole 3,3′-diindolylmethane), a compound known for its potential to target CSC-induced EMT. Both compounds were delivered effectively to the tumor cells using mesoporous silica nanoparticles encapsulated in exosomes (e-DDMSNP), which enhanced the stability and specificity of the treatment. This novel approach led to a significant reduction in DOX-induced EMT in TNBC cells [200], suggesting a promising treatment strategy for TNBC.

Other studies have investigated the role of TAMs in the progression of TNBC, focusing on their contribution to EMT and CSC properties. It was found that a high infiltration of the so-called anti-inflammatory, pro-tumor TAMS (also known as M2-like TAMs) in TNBC tissues, correlated with poor patients’ prognosis. The conditioned medium from those macrophages was shown to significantly promote EMT and CSC traits in TNBC cell lines (BT549 and HCC1937). The mechanism involved the secretion of chemokine CCL2 (C-C motif ligand 2) by TAMs, which activated AKT signaling and led to the nuclear translocation of β-catenin. Inhibition of β-catenin reversed the TAM-induced EMT and CSC properties, suggesting that the CCL2/AKT/β-catenin signaling pathway plays a crucial role in TAM-driven tumor progression [201].

CSCs are characterized by high expression of ABC transporters, particularly ABCG2 (ATP-binding cassette sub-family G member 2), which contribute to their resistance against a wide range of cytotoxic drugs [202]. ABC transporters are a family of integral membrane proteins that actively transport various molecules across cell membranes thanks to the energy from ATP hydrolysis [203]. This includes the efflux of toxic substances like chemotherapeutic agents outside the cell. For instance, ABCG2 is identified as a key marker for the side population of CSCs responsible for TNBC initiation, resistance to therapy, and recurrence [204].

#### 4.2.2. Increased Drug Efflux

Cancer cells develop the ability to evade the effects of a wide range of structurally and functionally different chemotherapeutic agents by the phenomenon called multidrug resistance (MDR). One mechanism of multidrug resistance involves the enhanced removal of drugs from cancer cells, a process driven by ATP-binding cassette (ABC) transporters [205]. These energy-dependent proteins, initially discovered in the 1970s [206,207], apart from their primary anticancer efflux capacity, are also involved in cancer pathogenesis and treatment response [208]. The dysregulation of ABC transporters impacts cancer cell survival, promotes tumor development, and contributes to cancer metastasis [209]. Moreover, studies have shown that ABC transporters’ expression is closely linked to the EMT process. EMT-inducing TFs, including Twist, Snail, and FOXC2, directly regulate ABC transporter genes, suggesting a molecular basis for the association between MDR and cancer invasiveness [210].

The key transporters implicated in drug efflux include MRP1 (multidrug resistance- associated protein-1; P-glycoprotein; ABCB1), which confers resistance to agents like vinca alkaloids and anthracyclines but not paclitaxel, MDR1 (multidrug resistance protein 1; ABCC1), which transports out of the cell a wide range of chemotherapeutics, including paclitaxel, and ABCG2 (also known as breast cancer resistance protein/BCR), responsible for the efflux of drugs such as DOX [167]. Drug resistance arises from the activity of these transporters, which by reducing the intracellular concentration of anticancer drugs, hinder their therapeutic effects, leading to diminished efficacy and treatment resistance [211]. Notably, the expression of ABC transporters is subtype-specific in BC, with increased levels often observed also in TNBC. While ABCB1 expression has been linked to metastatic progression and may serve as a marker for metastatic spread, ABCG2 expression in TNBC has unexpectedly been associated with improved clinical outcomes, suggesting its potential relevance in TNBC treatment strategies [212]. As aforementioned, TFs such as Twist, Snail, and FOXC2, when overexpressed in BC, enhance the promoter activity of ABC transporters, indicating that EMT regulates this transport [210].

Recent studies in TNBC have investigated the combined targeting of EGFR and Wnt/β-catenin signaling pathways to address metastasis and MDR, which are often driven by increased drug efflux. It was found that targeting EGFR alone with inhibitors like erlotinib or lapatinib was ineffective in TNBC, with low-dose lapatinib actually promoting EMT and MDR, thereby facilitating metastasis. However, co-targeting EGFR and Wnt/β-catenin pathways using lapatinib and a small molecule tankyrase inhibitor XAV939 led to MET, significantly enhancing the expression of epithelial marker E-cadherin and decreasing stemness marker EpCAM (epithelial cell adhesion molecule). This also reduced the expression of key drug efflux transporters, ABCB1 and ABCG2, highlighting the potential of the combinatorial targeting approach in overcoming MDR and limiting TNBC progression [65].

Interestingly, oligonucleotide aptamers, including the anti-EGFR CL4 aptamer, have also emerged as a promising EGFR targeting alternative in TNBC. When tested on aggressive EGFR-positive TNBC cell lines (BT-549 and MDA-MB-231), CL4 specifically inhibited vasculogenic mimicry (VM) and disrupted previously formed channels, an effect not seen with traditional EGFR inhibitors (erlotinib or cetuximab). Notably, CL4 impaired the interaction between integrin αvβ3 and EGFR, which is crucial for integrin αvβ3-dependent adhesion and VM formation. These data suggest a synergistic relationship between EGFR and integrin αvβ3, and highlight the potential of anti-EGFR CL4 aptamer as a novel therapeutic strategy [213]. Following treatment advancements related to integrins, other studies have also investigated integrin αvβ3-specific strategies, such as ProAgio, a protein that binds outside the classical ligand-binding site and induces apoptosis in integrin αvβ3-expressing cells [214]. These approaches highlight the therapeutic potential of directly targeting integrins that could complement EGFR-targeting therapies in aggressive cancers like TNBC. It also emphasizes the importance of integrins in mediating drug resistance and TNBC tumor progression, positioning integrin-targeted therapies as valuable “tools” for overcoming the limitations of current TNBC treatments.

#### 4.2.3. Increased Resistance to Apoptosis

In TNBC, EMT plays a crucial role in promoting resistance to apoptosis, a key process for cell survival. This resistance is driven by the dysregulation of apoptotic proteins, enabling TNBC cells to evade programmed cell death (PCD). As a result, TNBC cells become more aggressive, and treatment failures are common in patients [167,215].

During EMT, TNBC cells undergo significant molecular alterations. These include the downregulation of pro-apoptotic proteins, such as Bax, and the upregulation of anti-apoptotic factors like Bcl-2, Bcl-xL, and Mcl-1 [216]. These changes create a cellular environment promoting survival by resisting apoptosis. Additionally, the expression of epithelial markers is downregulated, further promoting EMT and the transition from an epithelial to a mesenchymal phenotype in TNBC is associated with changes in cellular adhesion, migration, and resistance to apoptosis [217,218].

TGF-β, a key regulator of EMT, plays a key role in modulating resistance to chemotherapy in TNBC. Studies have shown that TGF-β significantly contributes to the resistance of TNBC cells to the chemotherapeutic agent epirubicin by regulating both EMT and apoptosis. Specifically, TGF-β induces EMT and impairs apoptotic processes, making these cells more resistant to drug-induced death [61]. Experimental studies using mammary gland cell lines have also shown that TGF-β1-induced EMT leads to increased resistance to apoptosis. The cells that underwent EMT exhibited a significantly lower degree of caspase-3 activation (a marker of apoptosis) compared to the control cells. The resistance to apoptosis was most prominent in cells that had fully acquired the mesenchymal phenotype, suggesting that the ability to resist apoptosis is a progressive characteristic that develops as EMT advances [219].

Another central signaling pathway involved in both EMT and apoptosis resistance in TNBC is the AKT pathway. In TNBC cells, a member of the inhibitor of apoptosis protein (IAP) family, cIAP2 (cellular inhibitor of apoptosis protein 2) has been identified as a key regulator of EMT through the activation of AKT. Activated AKT phosphorylates GSK3β (glycogen synthase kinase 3 beta), leading to the stabilization of the transcription factor Snail. Snail then represses E-cadherin expression, driving the transition toward a mesenchymal phenotype while simultaneously inhibiting apoptosis. Moreover, AKT activation also triggers the phosphorylation of IκBα (inhibitor of kappa B alpha), which enables the transcriptional activation of NF-κB, further promoting cancer cell survival [220].

The increased resistance to apoptosis in EMT is a critical factor in the survival of cancer cells, especially in the context of chemotherapy. This resistance complicates treatment, particularly in cancers such as TNBC that exhibit a high degree of EMT activity. Therefore, targeting IAP proteins, including cIAP2, presents an attractive therapeutic strategy. Inhibiting these proteins using small molecules, such as SMAC (second mitochondria-derived activator of caspases) mimetics, could enhance cancer cell sensitivity to apoptosis while simultaneously preventing further progression of EMT, offering a promising approach to improve treatment outcomes for TNBC patients [220].

#### 4.2.4. Enhanced DNA Damage Repair Mechanisms

DNA damage response (DDR) is a highly conserved and complex signaling network that senses DNA damage and enables a cascade of DNA repair mechanisms [221]. TNBC cells undergoing EMT possess great DNA repair capabilities and can upregulate DNA repair pathways, allowing them to tolerate DNA-damaging agents, e.g., platinum-based drugs (PBD) (recently reviewed in [222]). Enhanced DNA repair is a hallmark of EMT-mediated chemoresistance in TNBC, contributing to treatment failure in patients receiving genotoxic therapies.

The EMT process, particularly the TFs involved, has been also shown to be involved in the choice of DNA damage repair mechanisms. For instance, ZEB1 promotes DDR and directly influences the choice of DNA double-strand break (DSB) repair pathways by favoring homologous recombination (HR) over error-prone mechanisms like polymerase theta-mediated end joining (TMEJ) [223,224,225,226]. This function is supported by TGF-β signaling, which induces EMT-TF expression and enhances HR activity by suppressing miR-182, a repressor of BRCA1 and FOXO3 (forkhead box O3) [227]. Additionally, repair factors such as PARP1 (poly(ADP-ribose) polymerase 1) and DNA-PKcs (DNA-dependent protein kinase catalytic subunit) are associated with EMT regulation, influencing the stabilization and activity of EMT-TF like SNAIL, which further modulate DNA repair pathways [228,229].

The overexpression of RAD50 (radiation-sensitive 50 kDa protein), a crucial component of the HR repair pathway, enables TNBC cells to recover more effectively from chemotherapy by enhancing HR repair through the MRN (Mre11 and NBS1) complex [230]. RAD50 protein functions as a DSB sensor and activates ATM (ataxia–telangiectasia mutated) serine/threonine protein kinase and promotes DNA repair, cell cycle arrest, and apoptosis [231]. Other recent studies have shown that the expression level of METTL3 (methyltransferase-like 3) enzyme, involved in the modification of RNA molecules through a process called N6-methyladenosine (m6A) methylation, is higher in TNBC compared to other breast cancer subtypes and normal breast tissue (MCF-10A cells; a non-tumoral breast epithelial cell line) [232]. Importantly, METTL3 is recruited to DNA damaged sites, where it facilitates repair processes essential for maintaining genomic stability [233,234]. Inhibition of METTL3 catalytic activity using the small-molecule STM2457 has shown promise in sensitizing cancer cells to DNA-damaging therapies [235,236]. STM2457 treatment significantly reduced cell proliferation, migration, and colony formation in TNBC cell lines, with minimal effect on non-tumoral breast cells. METTL3 inhibition not only disrupts DNA repair but may also impair EMT-driven processes, potentially enhancing the sensitivity of TNBC to chemotherapy and DNA-damaging agents [232].

Integrin α6β4, known for its role in promoting progression, migration, invasion, and metastasis in TNBC, has also been implicated in modulating DDR pathways [78]. Chen et al. [78] reported that integrin α6β4 signaling enhances sensitivity to platinum-based chemotherapies, such as cisplatin and carboplatin, through its influence on DDR proteins, including ATM, p53, and 53BP1 (tumor suppressor p53-binding protein 1). In response to cisplatin-induced DNA DSB, integrin α6β4 suppressed the HR activity and enhanced non-homologous end joining (NHEJ) repair activity. Moreover, it led to the preferential DNA-dependent protein kinase (DNA-PK) activation and the formation of DNA-PK-p53 and p53-53BP1 complexes, further strengthening the DDR to platinum-induced DNA damage. These findings suggest integrin α6β4 as a critical mediator of DDR mechanisms in TNBC, highlighting its significance as a therapeutic target for improving platinum chemotherapy response [78].

#### 4.2.5. Evasion of Immune Surveillance

Cancer immune surveillance is a crucial mechanism via which the immune system detects, identifies, and eliminates tumor cells. According to the so-called “three Es” model (“elimination, equilibrium, and escape”), the immune system effectively eliminates malignant cell precursors and suppresses neoplastic growth until tumor cells acquire genetic or epigenetic changes that allow immune escape. This escape is facilitated by mechanisms of the “three Cs” framework, i.e., camouflage, coercion, and cytoprotection, which protect cancer cells from immune detection, attack, and elimination [237,238].

While not directly related to chemoresistance in the traditional sense, the EMT-mediated immune evasion contributes to the overall survival and persistence of TNBC cells in the TME. TNBC tumors with strong EMT signatures often exhibit increased expression of PD-L1 (programmed death-ligand 1; in approximately 20% of TNBC cases) protein, and immunosuppressive microenvironments, reducing treatment efficacy. Importantly, increased PD-L1 expression on the surface of TNBC cells led to reduced T-cell proliferation and facilitated T-cell apoptosis, thereby suppressing the immune response [239].

Kumar et al. [240] investigated the role of Crk (cytoplasmic receptor for kinase), an adaptor protein, in regulating immune evasion in TNBC. Using a murine 4T1 adenocarcinoma model, they showed that Crk knockout (KO) enhances anti-tumor immune responses by increasing cytotoxic immune cells and immune surveillance cytokines in the primary tumor. These result in reduced tumor growth and metastasis. Mechanistically, Crk KO suppresses EMT and PD-L1 expression on tumor cells, and its effects are additive with anti-PD1 therapy, improving overall anti-tumor efficacy. These findings suggest that Crk plays a key role in immune evasion in breast cancer and could serve as a potential therapeutic target to enhance immunotherapy outcomes [240]. Another study into BC models revealed that the plasticity of EMT contributes directly to the development of immunosuppressive characteristics. Notably, transgenic mice lacking certain partial EMT markers (CD73, CSF1, or SPP1) showed slower tumor growth progression and heightened responsiveness to anti-CTLA-4 (cytotoxic T-lymphocyte antigen 4) treatment [241].

## 5. Discussion

TNBC is characterized by the lack of expression of ER, PR, and HER2 receptors. In contrast to non-TNBC subtypes, TNBC is often associated with a more aggressive clinical course, worse patient outcomes, and minimal responsiveness to hormonal therapies [242]. EMT plays a critical role in driving the aggressive nature of this BC subtype by promoting tumor cell plasticity, immune evasion, and metastatic potential [118,243]. This aggressiveness is further exemplified by the high mutation frequency of *TP53* (~80%) in TNBC, which is consistent with prior studies reporting mutations in 65–80% of cases [38,39]. *TP53* mutations impair the tumor-suppressive functions of the p53 protein, affecting key processes such as cell cycle regulation, DNA repair, and apoptosis, ultimately contributing to TNBC’s poor prognosis. Additionally, our findings of a lower mutation frequency in *PIK3CA* (15%) in TNBC, compared to its higher frequency in other BC subtypes (40.3%), suggest that distinct molecular mechanisms drive TNBC. Recent findings by Zaikova et al. [244] highlight the utility of circulating tumor DNA (ctDNA) as a biomarker for detecting drug resistance in TNBC. In this study, ctDNA detection within seven months of completing primary treatment identified residual disease in 7.7% of patients. Notably, incomplete pathological response combined with positive ctDNA was associated with significantly reduced DFS in patients treated with neoadjuvant therapy [244]. These results suggest that integrating such ctDNA analysis could aid earlier identification of drug resistance in TNBC.

Based on our recent results from the TCGA database (Figure 3), we observed significant upregulation of genes encoding key EMT transcription factors, including *SNAIL1* and *ZEB1*, the EMT-related transcriptional regulators such as *FOXC2* and *NOTCH1*, as well as genes encoding EMT marker proteins *VIM*, *KRT18*, *KRT8* as well as *MMP9*, in TNBC compared to other breast cancer subtypes (nTNBC). Among these, transcription factors and regulators of transcription drive the EMT process by repressing epithelial markers and promoting mesenchymal gene expression [179,245,246]. The EMT marker proteins, such as VIM, KRT18, KRT8, and MMP9 are involved in cell structure, motility, and matrix remodeling, which are crucial for tumor invasion and metastasis [247]. The heatmap analysis, along with hierarchical clustering, revealed distinct molecular profiles that to some extent differentiate TNBC from nTNBC. The observed upregulation of transcription factors encoding genes (e.g., *SNAIL1* and *ZEB1*) and transcriptional regulators (e.g., *FOXC2* and *NOTCH1*) indicates that TNBC tumors are more likely to undergo EMT, while generally the activated mesenchymal marker genes are associated with a shift to a more invasive phenotype. For instance, the upregulation of *VIM* signifies a mesenchymal transition, which is associated with enhanced cell motility, invasiveness, and metastasis [118,248]. The dysregulation of cytokeratins *KRT18* and *KRT8* in TNBC may suggest that TNBC cells retain a hybrid epithelial–mesenchymal state rather than undergoing complete EMT, enabling them to balance structural stability and invasiveness, which is crucial for metastasis. Moreover, *MMP9* is important for degrading extracellular matrix components, facilitating tumor invasion and metastasis [249,250].

The findings presented in this review highlight the critical role of the EMT process in TNBC progression and suggest that targeting both EMT transcription factors and markers could offer promising therapeutic strategies to limit metastasis and improve patient outcomes. Importantly, EMT may also contribute to TNBC heterogeneity, emphasizing the need to investigate the detailed molecular mechanisms underlying this process.

## 6. Conclusions

TNBC is a highly aggressive subtype of BC, characterized by poor prognosis and limited treatment options. Despite identifying potential molecular targets, the development of effective therapies has been hindered by the complex and heterogeneous nature of TNBC, marked by phenotypic plasticity and the activation of compensatory signaling pathways that promote tumor survival, metastasis, and progression. As a result, chemotherapy remains the primary treatment, but its long-term efficacy is severely limited by acquired resistance.

A major driver of chemoresistance in TNBC is EMT, which contributes to metastatic potential and diminishes chemotherapy effectiveness by altering drug uptake or enhancing efflux mechanisms. Additional factors such as altered drug metabolism and the TME further complicate treatment, emphasizing the urgent need for novel, targeted, and personalized therapies.

To overcome chemotherapy resistance and improve patients’ outcomes, it is essential to address the molecular complexity of TNBC, particularly the interplay between EMT, drug resistance, and tumor progression. While substantial progress has been made in understanding the role of EMT, the precise molecular mechanisms driving this process and its impact on resistance still remain largely unknown. Targeting key EMT-related pathways offers promising strategies for developing more effective therapies. A deeper understanding of these mechanisms is thus crucial for designing personalized treatment approaches that enhance TNBC management, improve survival, and elevate patients’ quality of life.

## Figures and Tables

**Table 1:** Key factors that influence epithelial–mesenchymal transition (EMT) and their impact on TNBC.

Category	Factor	Examples	Impact on EMT	Role in TNBC	Source
Internal factors	Genetic mutations	e.g., in *TP53* gene	Increase the expression of EMT-promoting transcription factors (TFs)	Promotion of cancer cell migration and invasion; treatment resistance	[52]
	Transcription factors	Snail, Slug, Twist, ZEB1, ZEB2	Activation of genes involved in EMT and regulation of EMT-associated genes’ expression	Promotion of cancer cell migration and invasion	[53,54,55]
	Non-coding RNAs	Specific miRNAs, lncRNAs, circRNAs	Post-transcriptional regulation of EMT-related genes’ expression	TNBC progression; aberrant expression related to chemoresistance	[56]
	Cell–cell junctions	E-cadherin (downregulation), N-cadherin (upregulation), zonula occludens, claudins	EMT progression via disruption of adherens junctions and tight junctions	Promotion of cancer cell migration and invasion	[57]
	Epigenetic factors	Modifications of histones, DNA methylation, chromatin remodeling	EMT promotion and progression by alterations of EMT-related genes’ expression	Promotion of cancer cell migration, invasion and resistance to therapies	[58]
	Post-translational modifications	Phosphorylation, glycosylation, methylation, acetylation, ubiquitylation, sumoylation	Regulation of stability, localization and activity of EMT-related proteins	Promotion of cancer cell migration, invasion and resistance to therapies	[59,60]
External factors	Growth factors	TGF-β, EGF, HGF, FGF	EMT promotion by the activation of downstream signaling pathways	Promotion of cancer cell migration, invasion and resistance to therapies	[61,62]
	Cellular signaling pathways	Wnt/β-catenin, Notch, PI3K/AKT, MAPK	EMT promotion by the activation of signaling cascades	TNBC progression to more aggressive phenotype, therapy resistance	[63,64,65,66]
	Cytokines	IL-6, IL-8, TNF-α	EMT promotion by the activation of signaling pathways that upregulate EMT-related TFs	Enhancement of cancer cell migration, invasion, and metastasis	[67]
	Extracellular matrix (ECM)	Fibronectin, collagens, laminins	EMT promotion	Promotion of cancer cell migration and invasion	[68]
	Mechanical forces	Cell contractility (via RhoA, Rock1), stiffness of the ECM	Tumor initiation, invasion, migration, metastasis	Promotion of cancer cell proliferation and migration	[69]
	Hypoxia	HIF-1α activation	Induction of EMT-related genes’ expression	Promotion of cancer cell migration, invasion and metastasis	[70]

**Table 2:** The main signaling pathways involved in EMT mechanisms, progression, and drug resistance in TNBC.

Signaling Pathway in the Context of EMT in TNBC	Pathway Involvement in EMT and/or Impact on TNBC	Source
TGF-β	EMT induction, promotion of invasion, metastasis, and drug resistance in TNBC	[136]
Aurora A/TGF-β	EMT regulation, with implications for tumor progression and drug resistance in TNBC	[137]
NF-κB	EMT promotion by TF induction; promotion of metastasis; therapy resistance in TNBC; and enhancement of pro-survival and pro-inflammatory cytokines	[138]
Notch1	EMT regulation, with implications for tumor progression and drug resistance in TNBC	[139]
Wnt	EMT enhancement by the activation of β-catenin; promotion of cell migration and invasion	[140,141]
PI3K/AKT/mTOR	EMT induction; promotion of migration and invasion of TNBC cells	[142]
MAPK	EMT involvement; metastatic potential enhancement	[142]
Hippo	EMT modulation through the regulation of YAP/TAZ; promotion of CSC properties	[143,144]
JAK/STAT3	EMT involvement; linked to migration, invasion, and metastasis as well as poor TNBC patients’ prognosis	[145,146]
p53	EMT inhibition by suppressing ZEB1 and ZEB2	[147]
GSK3β	EMT and CSCs properties regulation, poor patients’ survival, and drug resistance in TNBC	[148]

## Data Availability

The R code used to generate Figure 2 and Figure 3 is available upon request. This study utilized previously published data from the TCGA-BRCA dataset. The TCGA-BRCA RNA-seq dataset was downloaded from the GDC Data Portal (based on GDC data release 12) using genome reference build GRCh38.p0 (https://portal.gdc.cancer.gov (accessed on 16 November 2024)) with TCGAbiolinks. Mutation data were sourced from the Broad Institute TCGA Genome Data Analysis Center (2016) and generated using MutSig 2CV v3.1 [82].

## Abbreviations

ABC	ATP-binding cassette
ALDH1	Aldehyde dehydrogenase 1
AR	Androgen receptor
BC	Breast cancer
BLIA	Basal-like immune-activated
BLIS	Basal-like immunosuppressed
CAFs	Cancer-associated fibroblasts
CHK1	Checkpoint kinase 1
circRNA	Circular RNA
CK	Cytokeratin
CLDNs	Claudins
CSCs	Cancer stem cells
CTCs	Circulating tumor cells
DDR	DNA damage response
DFS	Disease-free survival
ECM	Extracellular matrix
EGFR	Epidermal growth factor receptor
EMT	Epithelial–mesenchymal transition
ER	Estrogen receptor
FAK	Focal adhesion kinase
HER2	Human epidermal growth factor 2
HIF-1α	Hypoxia-inducible factor 1-alpha
HR	Hormone receptor
IHC	Immunohistochemistry
IM	Immunomodulatory
LAR	Luminar androgen receptor
LEF	Lymphoid enhancer factor
LI	Labeling index
lncRNA	Long non-coding RNA
M/MES	Mesenchymal
MAPK	Mitogen-activated protein kinase
MDR	Multidrug resistance
MET	Mesenchymal–epithelial transition
miRNA	MicroRNA
MMP	Matrix metalloproteinase
MSL	Mesenchymal stem-like
mTOR	Mechanistic target of rapamycin
NACT	Neoadjuvant chemotherapy
ncRNA	Non-coding RNA
NF-κB	Nuclear factor kappa-B
OS	Overall survival
PCD	Programmed cell death
pCR	Pathological complete response
p-EMT	Partial epithelial–mesenchymal transition
PR	Progesterone receptor
PTMs	Post-translational modifications
QNBC	Quadruple-negative breast cancer
RCB	Residual cancer burden
TCF	T-cell factor
TCGA	The Cancer Genome Atlas
TF	Transcription factor
TGF-β	Transforming growth factor-beta
TJ	Tight junction
TNBC	Triple-negative breast cancer
TNF-α	Tumor necrosis factor α-induced
VIM	Vimentin
Wnt	Wingless/Integrated
ZO-1	Zonula occludens-1
